# Effect of High-Intensity Interval Training on Body Composition, Cardiorespiratory Fitness, Blood Pressure, and Substrate Utilization During Exercise Among Prehypertensive and Hypertensive Patients With Excessive Adiposity

**DOI:** 10.3389/fphys.2020.558910

**Published:** 2020-10-19

**Authors:** Pedro Delgado-Floody, Mikel Izquierdo, Robinson Ramírez-Vélez, Felipe Caamaño-Navarrete, Roberto Moris, Daniel Jerez-Mayorga, David C. Andrade, Cristian Álvarez

**Affiliations:** ^1^Department of Physical Education, Sport and Recreation, Universidad de La Frontera, Temuco, Chile; ^2^Navarrabiomed, Complejo Hospitalario de Navarra (CHN)-Universidad Pública de Navarra (UPNA), IdiSNA, Pamplona, Spain; ^3^CIBER of Frailty and Healthy Aging (CIBERFES), Instituto de Salud Carlos III, Madrid, Spain; ^4^Grupo GICAEDS, Facultad de Cultura Física, Deporte y Recreación, Universidad Santo Tomás, Bogotá, Colombia; ^5^Faculty of Education, Universidad Católica de Temuco, Temuco, Chile; ^6^Faculty of Rehabilitation Sciences, Universidad Andres Bello, Santiago, Chile; ^7^Centro de Investigación en Fisiología del Ejercicio, Universidad Mayor, Santiago, Chile; ^8^Quality of Life and Wellness Research Group, Department of Physical Activity Sciences, Universidad de Los Lagos, Osorno, Chile

**Keywords:** obesity, hypertension, blood pressure, metabolic flexibility, cardiorespiratory fitness

## Abstract

Regular exercise training is a recognized lifestyle strategy to lower resting blood pressure (BP), but little is known about substrate metabolism in population with high BP. Thus, the purpose of this study was to investigate the effects of 16-weeks of HIIT on body composition, BP, cardiorespiratory fitness by $\dot{V}$O_2__*max*_, and substrate utilization during exercise among prehypertensive and hypertensive patients with excessive adiposity. We also aimed to test the potential association between changes in cardiorespiratory fitness, substrate utilization during exercise and BP. Forty-two physically inactive overweight/obese participants participated in 16-weeks of HIIT intervention. The HIIT frequency was three times a week (work ratio 1:2:10, for interval cycling: rest period: repeated times; 80–100% of the maximum heart rate). Groups were distributed based on their baseline BP: HIIT-hypertensive (H-HTN: age 47.7 ± 12.0 years; body mass index [BMI] 30.3 ± 5.5 kg/m^2^; systolic [SBP]/diastolic BP [DBP] 151.6 ± 10/81.9 ± 4.2 mmHg), HIIT-pre-hypertensive (H-PreHTN: age 37.6 ± 12.0 years; BMI 31.9 ± 5.3 kg/m^2^; SBP/DBP 134.4 ± 3.2/74.9 ± 7.0 mmHg), and a normotensive control group (H-CG: age 40.7 ± 11.0 years; BMI 29.5 ± 4.2 kg/m^2^; SBP/DBP 117.0 ± 6.2/72.4 ± 4.1 mmHg). Anthropometry/body composition, BP, and metabolic substrate utilization during exercise (fat [FATox], carbohydrate [CHOox] oxidation, respiratory exchange ratio [RER], and $\dot{V}$O_2__max_), were measured before and after the 16-week HIIT intervention. Adjusted mixed linear models revealed a significant improved in $\dot{V}$O_2__max_ were + 3.34 in the H-CG, + 3.63 in the H-PreHTN, and + 5.92 mL⋅kg^–1^⋅min^–1^, in the H-HTN group, however, the Time × Group interaction were not significant (*p* = 0.083). All the exercise types induced similar decreases on SBP (−8.70) in the H-HTN, (−7.14) in the H-CG, and (−5.11) mmHg in the H-PreHTN, as well as DBP levels (−5.43) mmHg in H-CG group (*p* = 0.032 vs. H-HTN group). At 16-week, no significant correlations were noted for the changes of blood pressure, cardiorespiratory fitness or exercise metabolism substrates outcomes. In conclusion, our results suggest that a 16-week HIIT-intervention improved $\dot{V}$O_2__max_ and blood pressure BP, but these changes are independent of substrate utilization during exercise in normotensive and hypertensive participants with excessive adiposity.

## Introduction

Hypertension is the most common primary cardiometabolic disease in several Latin-American countries, with a prevalence of 27.7% in 2019 (Petermann et al., 2017). Not adhere to the international physical activity (PA) recommendations [i.e., 150 min/week of low/moderate PA, or 75 min/week of vigorous PA (O’Donovan et al., 2010)], is one of the most important factors for type 2 diabetes (T2DM) and other cardiometabolic disorders such as arterial hypertension (HTN), and has been associated with comorbidities including obesity and dyslipidaemia [i.e., higher low-density lipoprotein, total cholesterol or triglycerides (Russo et al., 2018)]. HTN is more prevalent in physically inactive populations (Diaz-Martinez et al., 2018), and it has long been recognized that untreated HTN might be linked to overweight/obesity, albuminuria and micro- and macrovascular changes including endothelial dysfunction and heart failure (Yannoutsos et al., 2014). Interestingly, chronic high blood pressure, such as HTN diagnosed, is related to several detrimental vascular effects (i.e., left ventricular hypertrophy, neurogenic dysfunction), which can affect patients systemically (Falqui et al., 2007; Diaz-Martinez et al., 2018). In these patients, and although not clear, the presence of some of these circulatory damage could also potentially have an impact on the normal metabolic and cardiorespiratory performance response during exercise (Neubauer, 2007). Additionally, defects in skeletal muscle lipid metabolism have been found in obese individuals during resting conditions and are associated with insulin resistance and T2DM (Goodpaster et al., 2002). Thus, it is speculative that HTN patients could respond in minor capacity (i.e., after long-term exercise training) than normotensive peers at metabolic behavior during exercise, however, there is limited information.

Exercise training (with characteristics of being controlled and periodized by intensity, volume, frequency and density) is highly recommended as a non-pharmacological treatment for HTN (Pescatello et al., 2015). Exercise training promotes angiogenesis in the skeletal muscles [i.e., the extension of vasculature from pre-existing micro-vessels (Kissane and Egginton, 2019)] and micro-circulation is a relevant adaptive mechanism that can contribute to exercise substrate metabolism and oxygen availability during effort (Hoppeler and Weibel, 1998). Thus, considering these exercise benefits at circulatory system, and given that HTN patients usually show a low micro-circulation (Feihl et al., 2006), there is a potential benefits from exercise training in patients with HTN that have been not at all elucidated.

There is growing evidence to suggest that high-intensity interval training [HIIT, defined as several, brief bouts of high-intensity efforts, usually via cycling/running, interspersed with recovery periods (Gibala et al., 2012)], promote similar adaptations, and in a time-efficient way than to continuous, moderate-intensity training for improving cardiorespiratory fitness (Costa et al., 2018), but with little advantage for improving vascular function (Ramos et al., 2015; Pedralli et al., 2020). Thus, HIIT might have protective effects against the development of HTN (Pescatello et al., 2015; Álvarez et al., 2018), and can lead to the reversal of a clinical diagnosis of HTN to prehypertension [PreHTN], or from PreHTN to normotension, in a relevant proportion of patients (Cano-Montoya et al., 2016; Álvarez et al., 2018). These beneficial cardioprotective effects have been more reported for long-term (i.e., >12 weeks) rather than short-term exercise programs (Jurio-Iriarte and Maldonado-Martín, 2019).

In the context of obesity, as a relevant HTN comorbidity and a target for exercise, fat oxidation rates have been found to be highest during low- to moderate-intensity exercise [i.e., MICT, moderate x watts of power output cycling (Achten et al., 2002)]. Intriguingly, the major mechanisms of adiposity loss associated with HIIT seem to be related to mitochondrial adaptations in skeletal muscle after HIIT, including an increase in mitochondrial biogenesis, and other molecular adaptations at Kreb’s cycle (into mitochondria) as increases in proteins citrate synthase, cytochrome oxidase, or at membranes proteins as fatty acid binding protein (FABP_*pm*_), or fatty acid CD36 (FAT/CD36) among others (Gibala et al., 2012; Astorino and Schubert, 2018). Also, the specific turn-on/turn-off periods in each interval bout in HIIT promote superior hormonal activity [i.e., adrenaline/noradrenaline catecholamines (Boutcher, 2010), and natriuretic peptide (Birjandi et al., 2016)] after exercise than traditional MICT modalities.

In this line, manipulation of exercise intensities (e.g., HIIT combined with resistance training or low- and MICT), however, does not seem to influence whole-body fat oxidation (Romijn et al., 1995). Also, a higher percentage of body fat does not necessarily translate into a greater FATox during exercise (Friedlander et al., 1999). Interestingly, an increased fat oxidation after circuit-type resistance training interventions has been observed in patients with impaired glucose tolerance, suggesting that other mechanisms may be involved (Eriksson et al., 1998). Thus, there is limited information on the effects of HIIT on blood pressure and metabolic substrate use such as FATox and CHOox in patients with HTN. Obesity is also characterized by skeletal muscle with a reduced transport and/or phosphorylation of glucose, leading to lower rates of fatty acid oxidation at rest, compared with lean insulin-sensitive individuals (Goodpaster et al., 2002). In the same line, the rate of muscle glycogen oxidation during exercise is reduced in obese patients despite rates of plasma glucose uptake that are similar to lean controls. However, little is known regarding patterns of fuel use during exercise in hypertension patients with obesity, and there are no studies that compare the effects of different exercise training methodologies in this population.

Therefore, designing and evaluating an individualized exercise training program on the basis of the pattern of energy substrate utilization for treating or preventing overweight and obesity in HTN patients remains a critical task for exercise scientists. Thus, the purpose of this study was to investigate the effects of 16-weeks of HIIT on body composition, BP, cardiorespiratory fitness by $\dot{V}$O_2__max_, and substrate utilization during exercise among prehypertensive and hypertensive patients with excessive adiposity. Furthermore, we aimed to test the association between potential changes in $\dot{V}$O_2__max_, substrate utilization during exercise, and blood pressure. We hypothesized that BP improvements can be also associated with substrate utilization (i.e., fat and CHOox) during exercise in HTN patients.

## Materials and Methods

### Study Participants

Forty-two (female *n* = 21; male *n* = 21) physically inactive adults (non-adherent to 150 min/week of low to moderate physical activity (PA)/week, or to 75 min/week of vigorous PA (O’Donovan et al., 2010), screened by the international physical activity questionnaire (IPAQ) (Serón et al., 2010), with or without hypertension were recruited. Participants were invited to participate in an exercise intervention programme at the Universidad de La Frontera Exercise Laboratory, Chile through open information disseminated by the research center (i.e., social network and email). All participants signed a written informed consent form which complied with the requirements of the last revised Declaration of Helsinki and was approved by the Human Research Ethics Committee of the Universidad de La Frontera, Chile (DI18-0043).

The inclusion criteria were as follows: (i) to have diagnosed clinical stage 1 or 2 hypertension, elevated blood pressure, or to be normotensive (see criteria classification below); (ii) to be adult >18 and <60 years of age (65 years is the retirement age for women in Chile); (iii) participation in physical autonomous daily activities including walking; (iv) medical authorization by a physician to take part in the study, and; (v) body mass index (BMI) ≥ 25 kg/m^2^ ≤ 39.9 kg/m^2^. *Exclusion criteria* were; (i) physical limitations (e.g., restricting injuries of the musculoskeletal system such as osteoarthritis, or to be dependent on a third person); (ii) exercise-related dyspnea or respiratory alterations; (iii) chronic heart disease; (iv) altered ECG, and; (v) an adherence rate <80%, in which case data were not included in the final statistical analysis.

**FIGURE 1:**
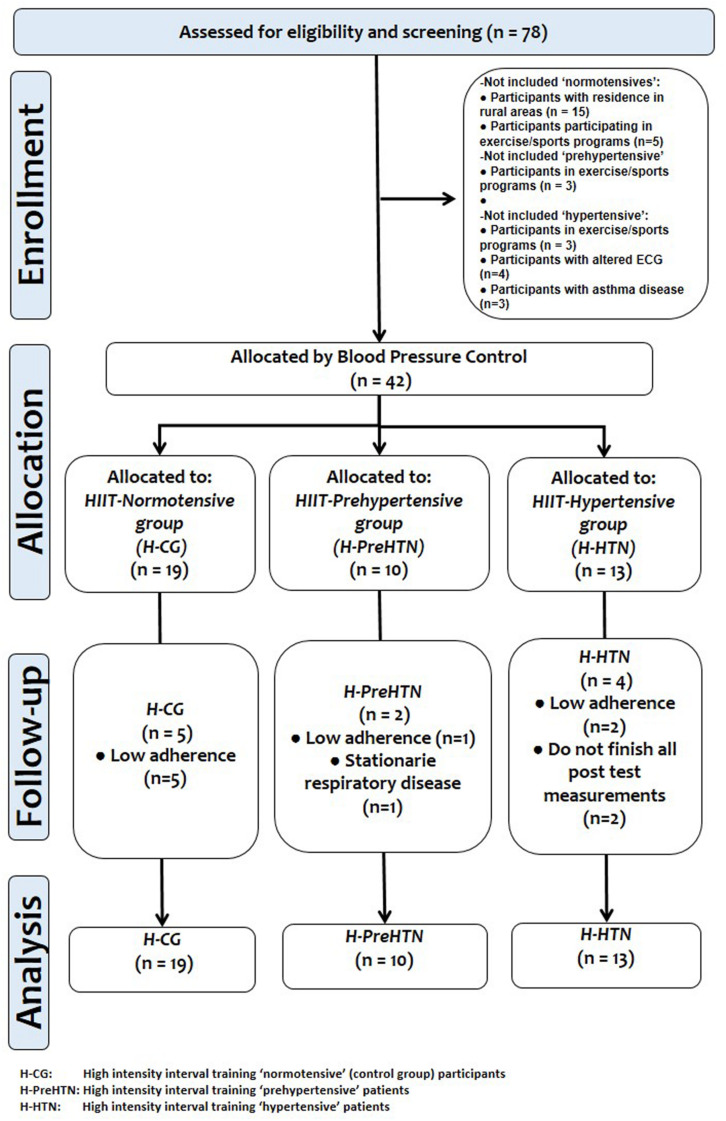
Flow-chart diagram.

A total of 42 patients were included in the final analysis. Participants were allocated to one of the three following groups according to their blood pressure (diagnosed by physician): HIIT-hypertensive (H-HTN: age 47.7 ± 12.0 years; body mass index [BMI] 30.3 ± 5.5 kg/m^2^; systolic [SBP]/diastolic BP [DBP] 151.6 ± 10/81.9 ± 4.2 mmHg), HIIT-pre-hypertensive (H-PreHTN: age 37.6 ± 12.0 years; BMI 31.9 ± 5.3 kg/m^2^; SBP/DBP 134.4 ± 3.2/74.9 ± 7.0 mmHg), and a normotensive control group (H-CG: age 40.7 ± 11.0 years; BMI 29.5 ± 4.2 kg/m^2^; SBP/DBP 117.0 ± 6.2/72.4 ± 4.1 mmHg). The groups were submitted to a 3 × weekly HIIT program for 16 weeks. The sample size was calculated using the G^∗^Power 3 Software (Faul et al., 2007). We used both delta changes and SD from previous studies of the similar exercise intervention extension, and cohort (patients with obesity), where blood pressure was included (Delgado-Floody et al., 2019). Based on this, with 1 predictive outcome (systolic BP [SD: 6 mmHg]), a moderate effect size (0.60) and a critical *t* value of 1.73, a total sample size of (*n* = 10) subjects per group would give a statistical power of 80%, under an alpha error *p* < 0.05. Flow-chart diagram is detailed in Figure 1.

### Blood Pressure and Anthropometric Measurements

Blood pressure was measured in the sitting position after 5 min rest. Two recordings were made, and the mean of the measurements was used for statistical analysis with an OMRON^®^ digital electronic BP monitor (model HEM 7114, Chicago, IL, United States). There was a 15-min rest interval between readings, as for previous exercise training studies on the cohort profile (Guimaraes et al., 2010). We used the currently published standard cut-off classification for BP of the American Heart Association by Whelton et al. (2018) considering the 4-categories; (i.e., *normal* [SBP < 120; DBP < 80 mmHg], *elevated* [SBP 120–129; DBP < 80 mmHg], *hypertension* stage 1 [SBP < 130–139; DBP 80–89 mmHg] and *hypertension* stage 2 [SBP ≥ 140; DBP ≥ 90 mmHg]), that is shown in (Figure 4B) in continuous and intermittent lines (left side). However, as these criteria have not been updated in the public health systems of some countries, and only for a major contrasting between this current, and the past criteria, we also included the ranges of the past traditional BP classification that included 3-categories; (i.e., normotension [NT], prehypertension [PreHTN], and hypertension [HTN]) from Chobanian et al. (2003), that we denoted in the same (Figure 4B) in color boxes, right side, and ranges limits to each category denoted by the same continuous/intermittent lines.

Body mass (kg) was measured using a digital bio-impedance scale (TANITA^®^, model Scale Plus UM – 028, Tokyo, Japan) and height (m) was measured with a SECA^®^ stadiometer (model 214, Hamburg, Germany), with subjects in light clothing and without shoes. Body mass index (BMI) was calculated as the body weight divided by the square of the height (kg/m^2^) and was used to estimate nutritional status.

### Cardiorespiratory Fitness, Indirect Calorimetry and Substrate Utilization During Exercise Calculations

All participants were also asked to avoid excessive physical activity, especially weight training and high-intensity exercise, to abstain from alcohol, caffeine, adrenergic beverages, consume fat, or nicotine for 8–10 h prior to blood pressure and anthropometrics measurements. On the morning of day 2, all participants returned to the laboratory between 7 and 9 am (72 h after the last exercise session), in the same order and with the same professional staff as in the baseline assessment. The maximal oxygen uptake ($\dot{V}$O_2__max_) was evaluated using the Åstrand test (Åstrand et al., 2003). The test is progressive, volitional and applied according to sex. The test starts with a rest period of 2 min, followed by 1 min of pedaling on a cycle ergometer (Lode^®^ model Corival, Groningen, The Netherlands) without load, and then the load is increased by 50 watts (for men) or 25 watts (for women) every 2 min, with a pedaling frequency of 60–70 revolutions per minute to achieve maximum cardiorespiratory fitness for estimation of $\dot{V}$O_2__max_ (Mancilla et al., 2014; Olea et al., 2017), where the results were expressed in mL⋅kg^–1^⋅min^–1^.

We also registered exercise substrate metabolism fat (FATox) and carbohydrate (CHOox) energy substrates] during the test by indirect calorimetry gas analysis (Ultima CPX Medgraphics^®^, St Paul, MN, United States), to measure oxygen consumption ($\dot{V}$O_2_) and carbon dioxide production ($\dot{V}$CO_2_), which were used to calculate rates of total lipid and carbohydrate oxidation, and the respiratory exchange ratio (RER). Systemic FAT and CHO oxidation rates were calculated using the stoichiometric equations of Frayn (1983). The relative contributions of FATox and CHOox to energy expenditure were calculated using the following equation (McGilvery and Goldstein, 1983):

% FAT = [(1–RER)/0.29] × 100

%CHO = [(RER–0.71)/0.29] × 100

**FIGURE 2:**
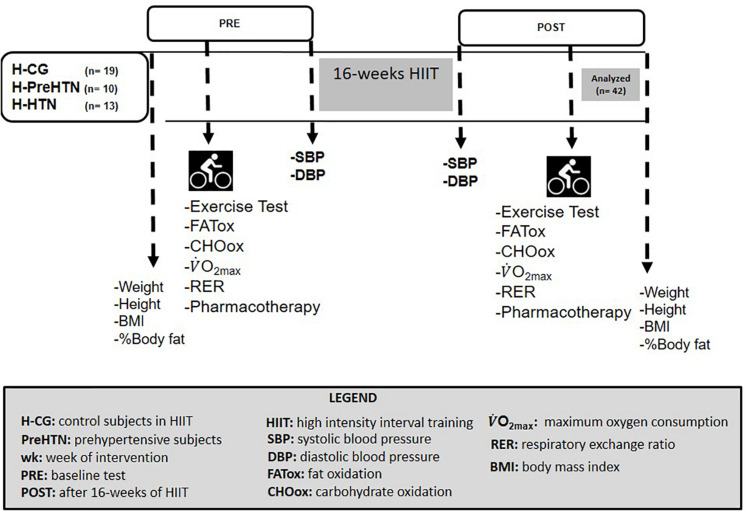
Study protocol.

The criteria for interruption and cessation of the exercise test were as follows: (a) a > 1.1 RER, (b) revolutions per minute (rpm) of ≤ 50 rpm (participants were recommended to exercise between 50–70 rpm), or (c) heart rate stabilization. The gas analyzers were calibrated with a certified calibration gas (4.95% CO_2_–95.05% O_2_, balance N_2_), and the volume transducer was calibrated with a 3-liter calibration syringe (Ultima CPX Medgraphics^®^, St Paul, MN, United States), before each test by a lab assistant. The study protocol is shown in Figure 2.

### Exercise Intervention

The HIIT intervention was performed with 1 min of maximum intensity exercise using a magnetic resistance static bicycle (Oxford^®^ Fitness, model BE-2701, Chile) followed by 2 min of passive recovery over the bicycle (i.e., no pedaling), and this was repeated 10 times (Mancilla et al., 2014). The HIIT frequency was three times a week. The intensity of the exercise was calculated by the heart rate (HR) obtained from the calorimeter with a workload of 8, 9, or 10 (high level) on the Borg scale of 1 to 10 of perceived exertion, which was different in terms of load in watts among participants, but of similar intensity by the modified Borg scale [8, 9, or 10 points (Gibala et al., 2012)]. Thus, the subjective intensity of cycling also corresponded to 80–100% of the maximum heart rate in each participant that was correlated with the aforementioned modified Borg scale, at 8–10 points. All participants received 3 previous exercise sessions of familiarization. A total 480 min of effective work and 960 min of pause (passive, no pedaling) were developed in the HIIT intervention.

### Statistical Analyses

The study data were processed using SPSS for Windows, program version 23.0 (SPSS^®^ Inc., Chicago, IL) and graphs created using GraphPad Prism 8.0.2 software (GraphPad Software, San Diego, California). The null hypothesis was rejected at a level of significance of *p* < 0.05, and all statistical tests were two-tailed. The data are shown as mean and 95% confidence intervals (CIs) in tables and as least-squares means with 95% CIs in figures. The normal distribution of the data and the equality of variances was checked using the Kolmogorov-Smirnov test and Levene’s test, respectively. Analysis of variance or Chi-square test (X^2^) test as appropriate were used to analyze differences in outcomes between groups from baseline.

The effect of the intervention was performed using a per-protocol analysis. We used linear mixed-effects modeling for repeated measures over time using $\dot{V}$O_2__max_, blood pressure, and, indirect calorimetry outcomes as the dependent variable and effects for time, group (H-CG, H-PreHTN or H-HTN), and time by group interaction, with age, gender and BMI as covariates and an unstructured covariance matrix. Within the mixed model, we calculated 95% *Cis* and *P* values for 3 pre-specified intergroup contrasts and for change for all continuous variables within each group over time with adjustment for the baseline values, age, gender and BMI as covariates. A Sidack’s *post hoc* test was used for multiple comparisons. Eta partial squared for interaction (Time × Group) was assessed by η^2^ obtained from the ANCOVA with small (η^2^ = 0.01), medium (η^2^ = 0.06), and large (η^2^ = 0.14) effects defined according to Lakens (2013). Liner regression tests were used in order to investigate the correlations between ΔCRF and ΔBP, and between ΔBP and changes in exercise substrate metabolism outcomes (ΔFATox, ΔCHOox, and ΔRER) adjusted for age, gender and BMI.

## Results

Regarding anthropometric measures, there were no significant differences at baseline for the three groups in terms of body mass: H-CG 78.84 (95% CI, 73.35 to 84.41), H-PreHTN 90.70 (95% CI, 76.39 to 105.01) and H-HTN 84.64 (95% CI, 71.41 to 97.88) kg, *p* = 0.222), BMI: H-CG 29.50 (95% CI, 27.49 to 31.51), H-PreHTN 31.97 (95% CI, 28.19 to 35.75) and H-HTN 30.33 (95% CI, 28.82 to 31.84) kg/m^2^, *p* = 0.439, or body fat: H-CG 34.25 (95% CI, 31.50 to 37.06), H-PreHTN 35.72 (95% CI, 31.20 to 40.23) and H-HTN 34.33 (95% CI, 30.73 to 37.93) percentage, *p* = 0.801, Table 1. There were significant differences in the number/proportion of subjects diagnosed with hypertension at pre-test (13 [100%]) *versus* those at post-test (3 [23.0%]) (*p* < 0.001) (Table 1).

**TABLE 1:** Characteristics of normotensive, prehypertensive, and hypertensive participants of 16-weeks of high intensity interval training.

**Characteristics**	**Time**	**H-CG**	**H-PreHTN**	**H-HTN**	***p* value**
N		19	10	13	
Age (y)		40.74 (35.34 to 46.13)	37.60 (29.10 to 46.10)	47.77 (38.40 to 45.94)	*p* = 0.105^a^
***Anthropometric/Body composition***					
Height (cm)		163.74 (160.59 to 166.88)	167.70 (162.20 to 173.20)	165.08 (159.92 to 170.24)	*p* = 0.406^ a^
Body mass (kg)	Pre	78.84 (73.35 to 84.41)	90.70 (76.39 to 105.01)	84.64 (71.41 to 97.88)	*p* = 0.222^ a^
	Δkg	−1.76 (−3.15 to −0.37)	−1.99 (−3.91 to −0.07)	−2.79 (−4.49 to −1.08)	*p* = 0.661^*b*^
	*p* value	***p* = 0.032**	***p* = 0.010**	***p* = 0.002**	
Body mass index (kg/m^2^)	Pre	29.50 (27.49 to 31.51)	31.97 (28.19 to 35.75)	30.33 (28.82 to 31.84)	*p* = 0.439^ a^
	Δkg/m^2^	−0.67 (−1.27 to −0.06)	−0.77 (−1.61 to 0.05)	−0.39 (−1.13 to 0.35)	*p* = 0.769^*b*^
	*p* value	***p* = 0.041**	*p* > 0.05	*p* > 0.05	
Body fat (%)	Pre	34.25 (31.50 to 37.06)	35.72 (31.20 to 40.23)	34.33 (30.73 to 37.93)	*p* = 0.801^ a^
	Δ%	−0.71 (−1.63 to 0.20)	−1.17 (−2.43 to 0.09)	−1.38 (−2.50 to −0.26)	*p* = 0.642
	*p* value	*p* > 0.05	*p* > 0.05	***p* = 0.015**	
***Baseline Blood pressure***					
Systolic BP (mmHg)	Pre	117.01 (113.82 to 120.77)	134.40 (132.28 to 136.51)	151.69 (145.49 to 157.89)	***p* < 0.001**^ a^
Diastolic BP (mmHg)	Pre	72.42 (70.05 to 74.78)	74.90 (69.82 to 79.97)	81.76 (78,01 to 85.53)	***p* < 0.001**^ a^
***Substrate utilization during exercise (maximal)***					
Fat oxidation (%)	Pre	12.31 (4.81 to 19.81)	31.70 (17.35 to 46.04	26.69 (15.52 to 37.01)	***p* = 0.011**^ a^
CHO oxidation (%)	Pre	87.68 (80.18 to 95.18)	68.10 (53.93 to 82.70)	78.58 (62.93 to 83.52)	***p* = 0.011**^a^
Respiratory exchange ratio	Pre	0.98 (0.95 to 1.01)	0.89 (0.85 to 0.94)	0.91 (0.87 to 0.95)	***p* = 0.004**^a^
***Baseline Pharmacotherapy¶***					
Atenolol, *n* = (50 mg/1 uts. day)			–	13/13	
Losartan, *n* = (50 mg/1–2 uts. day)			1/10	8/13	
Hydrochlorothiazide, *n* = (12.5–25 mg/1 uts. day)			–	6/13	
Atorvastatin, *n* = (10–40 mg/1 uts. day)			5/10	1/13	
***Blood Pressure Diagnosed by Group***	Pre	19 (100%)	10 (100%)	13 (100%)	
	Post	29 (152.6%)	10 (100%)	3 (23.0%)	***p* < 0.001**^c^

*Data are shown in mean and 95% CIs to continuous outcomes, and as (*n/*percent to categorical outcomes). Groups are described as; (H-CG) HIIT-normotensive control group [Reference group], (H-PreHTN) HIIT-prehypertensive group, and (H-HTN) HIIT-hypertensive group. Bold values denotes significant differences within group, or inter-group at baseline at *p* < 0.05 or less. (¶) Pharmacotherapy is shown according with the proportion of participants per group taking it [e.g., 2 out of 13 (2/13)]. (uts.) Units. ^*a*^Analysis of variance in baseline. ^*b*^Analysis of covariance (ANCOVA) with adjustment for the baseline values, age, gender as covariates (*p*-value for Time × Group interaction). A Sidack’s *post hoc* test was used for multiple comparisons. ^*c*^Analysis by Chi-square test (X^2^) test.*

There were significant decreases in all groups for body mass: H-CG −1.76 (95% CI, −3.15 to −0.37) kg, *p* = 0.032; H-PreHTN −1.99 (95% CI, −3.91 to −0.07) kg, *p* = 0.010; and H-HTN −2.79 (95% CI, −4.49 to −1.08) kg, *p* = 0.002), however, the Time × Group interaction were not significant (*p* = 0.661) (Table 1). There were significant decreases in Δ BMI only in the H-CG group −0.67 (95% CI, −1.27 to −0.06) kg/m^2^, *p* = 0.041. Body fat (%) decreased in the H-HTN group −1.38 (95% CI, −2.50 to −0.26), *p* = 0.015 (Table 1).

**FIGURE 3:**
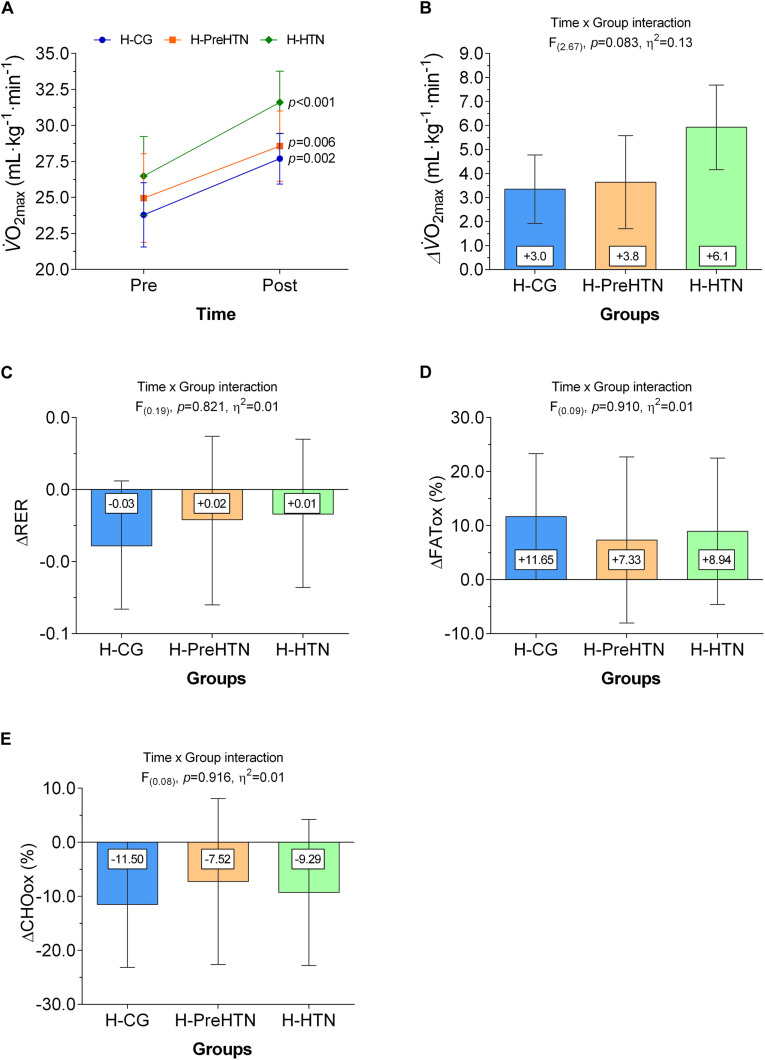
Maximum oxygen consumption ($\dot{V}$O_2__max_), respiratory exchange ratio (RER), FATox and CHOox utilization during exercise measured by indirect calorimetry measured in healthy normotensive, prehypertensive, and hypertensive subjects. Panel **(A)** show absolute values of $\dot{V}$O_2__max_, and Panel **(B)** show $\dot{V}$O_2__max_ in delta values pre-post 16-weeks HIIT intervention. Panel **(C)** show RER peak during exercise in delta values pre-post 16-weeks HIIT intervention. Panel **(D)** shows fat utilization during exercise in delta values pre-post 16-week high-intensity interval training (HIIT) intervention. Panel **(E)** shows CHO utilization during exercise in delta values pre-post 16-week HIIT intervention Groups are described as (H-CG) HIIT-normotensive control group, (H-PreHTN) HIIT-prehypertensive group, and (H-HTN) HIIT-hypertensive group. Within the mixed model, we calculated 95% *Cis* and *P* values for 3 prespecified intergroup contrasts and for change for all continuous variables within each group over time with adjustment for the baseline values, age, gender and BMI as covariates. A Sidack’s *post hoc* test was used for multiple comparisons. All results are presented as least-squares means with 95% confidence intervals (CIs).

Adjusted mixed linear models revealed a significant improved in $\dot{V}$O_2__max_ were + 3.34 (95% CI, 1.91 to 4.76) mL⋅kg^–1^⋅min^–1^ in the H-CG, + 3.63 (95% CI, 1.69 to 5.57) mL⋅kg^–1^⋅min^–1^ in the H-PreHTN, and + 5.92 (95% CI, 4.16 to 7.68) mL⋅kg^–1^⋅min^–1^ in the H-HTN group, however, the Time × Group interaction were not significant (*F*_(__2_._67__)_, *p* = 0.083, η^2^ = 0.13), Figures 3A,B.

At 16-week, RER and CHOox changes were not significantly different from baseline in the H-CG, H-PreHTN, or in the H-HTN group (*p* > 0.05) Figures 3C,E. The FATox increases in H-CG + 11.65 (95% CI, 0.02 to 23.33)%, but not in the H-PreHTN + 7.33 (95% CI, −8.04 to 22.71)%, or in the H-HTN group + 8.94 (95% CI, −4,61 to 22, 49)%, Figure 3D. However, the Time × Group interaction were not significant (*F*_(__0_._09__)_, *p* = 0.910, η^2^ = 0.01) (Figure 3D).

**FIGURE 4:**
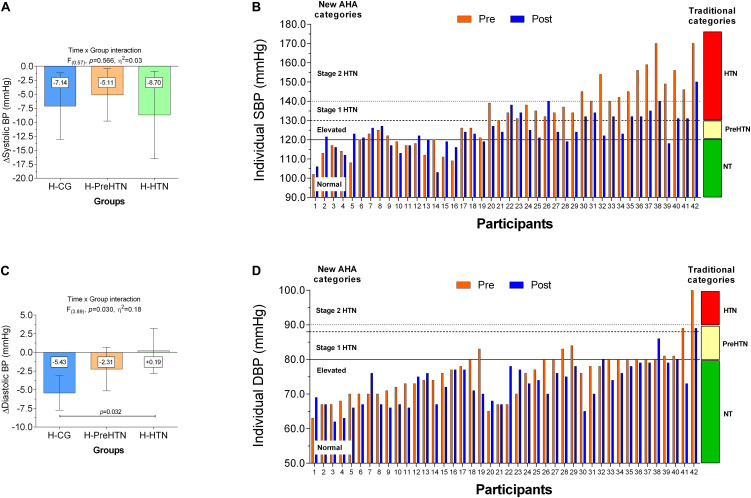
Systolic (SBP) and diastolic blood pressure (DBP) in healthy normotensive, prehypertensive, and hypertensive subjects. Panel **(A,C)** show SBP and DBP delta changes in mean values from pre to post interventions groups. Panels **(B,D)** show show SBP and DBP according with each pre-post individual change, and by the old traditional cut-off point from Chobanian et al. (2003) (continuous, very intermittent, and less intermittent black lines) of blood pressure diagnosed ranged in green, cream, and red boxes, right side **(B,D)**, as well as showing the new cut-off from Whelton et al. (2018) from the AHA 2017, **(B,D)**, left side. Groups are described as (H-CG) HIIT-normotensive control group, (H-PreHTN) HIIT-prehypertensive group, and (H-HTN) HIIT-hypertensive group.

SBP decreased in the H-HTN −8.70 (95% CI, −16.52 to −0.88) mmHg, H-CG −7.14 (95% CI, −13.10 to −1.18) mmHg, and H-PreHTN −5.11 (95% CI, −9.78 to −0.43) mmHg. No significant intergroup differences were observed (*F*_(__0_._57__)_, *p* = 0.566, η^2^ = 0.03), Figures 4A,B. DBP changes were −5.43 (95% CI, −7.73 to −3.12) mmHg in the H-CG group, and decreased significantly more in the H-HTN group (*p* = 0.032), Figure 4D.

**FIGURE 5:**
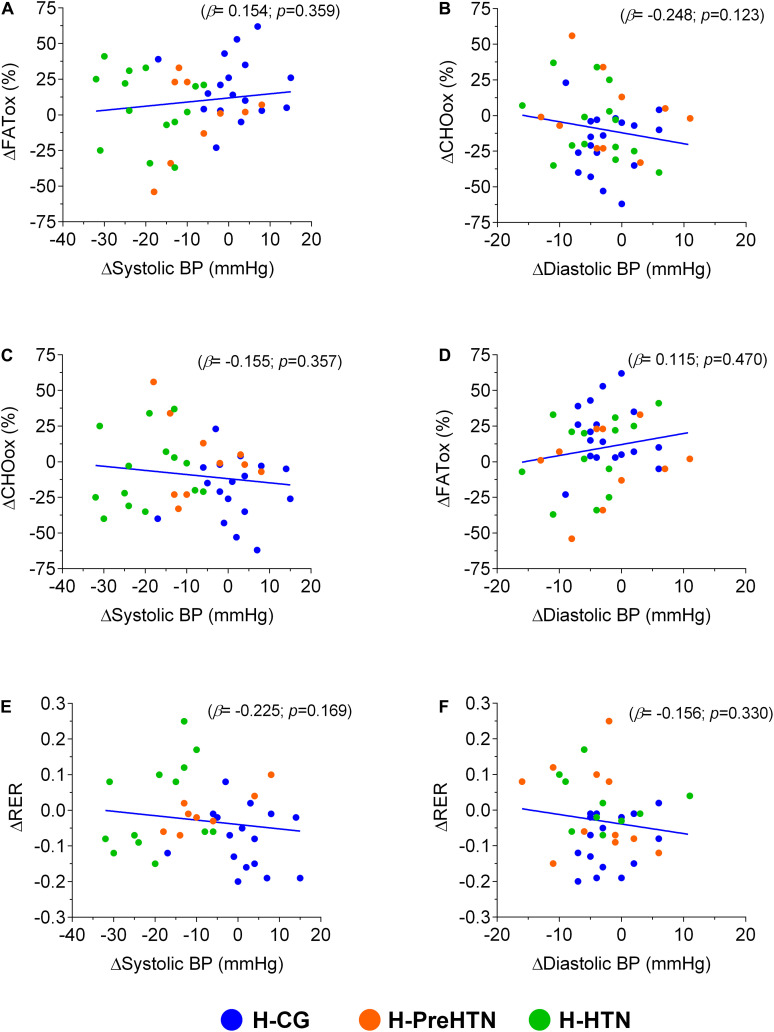
Correlations among the delta of fat (ΔFATox), delta of carbohydrate (ΔCHOox) oxidation, and delta of respiratory exchange ratio (ΔRER) with the delta of systolic (ΔSystolic BP), and diastolic (ΔDiastolic BP). Panel **(A)** show correlation ΔFATox with ΔSystolic BP. Panel **(B)** show correlation ΔCHOox with ΔDiastolic BP. Panel **(C)** show correlation ΔCHOox with ΔSystolic BP. Panel **(D)** show correlation ΔFATox with ΔDiastolic BP. Panel **(E)** show correlation ΔRER with ΔSystolic BP. Panel **(F)** show correlation ΔRER with ΔDiastolic BP. Groups are described as (H-CG) HIIT-normotensive control group, (H-PreHTN) HIIT-prehypertensive group, and (H-HTN) HIIT-hypertensive group. β = Standardized coefficients beta with adjustment for the age, gender and BMI as covariates. Both ΔFATox and ΔCHOox are expressed in %, respiratory exchange ratio (RER).

There were no significant correlations between the delta changes of blood pressure outcomes (ΔSBP/ΔDBP), the delta changes from the cardiorespiratory fitness (Δ$\dot{V}$O_2__max_, and ΔRER) and the delta changes for utilization during exercise outcomes (ΔFATox and ΔCHOox), Figure 5.

## Discussion

The main findings were that 16-weeks of HIIT protocol improved the $\dot{V}$O_2__max_, decreases the body mass and systolic BP especially in the pre-hypertensive and hypertensive patients, independent of changes in exercise substrate metabolism outcomes. Despite slightly greater improvements in some body composition variables or cardiorespiratory fitness, the changes observed in the exercise substrate metabolism were not related to the other BP changes.

The importance of exercise training for prevention and treatment of hypertension is well known (Cade et al., 1984; Guimaraes et al., 2010; Pescatello et al., 2015; Olea et al., 2017). Thus, part of the mechanisms by exercise training decrease blood pressure are explained and mediated by acute transitory mechanisms such as: (a) a reduction in vascular peripheral resistance (Ramírez-Vélez et al., 2019), (b) an increase in nitric oxide (Augeri et al., 2009), (c) a decrease in vasoconstrictors (Low et al., 2007), (d) an increase in shear stress (Birk et al., 2012), (e) a decrease in sympathetic nervous activity (Halliwill, 2001), and potentially other from structural chronic adaptations at vascular (Pedralli et al., 2020). For example, as the HTN patients decreased both SBP and DBP from the (Pedralli et al., 2020), where they also improved vascular structural parameters, from here it is possible to speculate that our results could be related with potential structural modifications. However, as we did not included vascular direct measurements, further research is needed to determine more clear these mechanisms.

In an exercise study on patients with hypertension (Molmen-Hansen et al., 2012) showed that 12-weeks of HIIT (4 × 4 min at 85–90% $\dot{V}$O_2__max_, walking/running, 38 min total session) or MICT (60% $\dot{V}$O_2__max_, walking/running, 47 min total session) reduced 24-h SBP by Δ−12mmHg (HIIT protocol) or Δ−4.5 mmHg (MICT protocol). Additionally, the authors showed that both exercise modes increased Δ$\dot{V}$O_2__max_ (HIIT Δ + 5.2 mL⋅kg^–1^⋅min^–1^, MICT Δ + 1.0 mL⋅kg^–1^⋅min^–1^), which is in accordance with our HIIT protocol in hypertensive patients (H-HTN Δ + 5.2 mL⋅kg^–1^⋅min^–1^). Unfortunately, they did not report on exercise metabolism outcomes or exercise substrate changes (Molmen-Hansen et al., 2012). We found that our low exercise-time/session HIIT protocol not only decreased SBP, but also led to relevant clinical changes in the proportion of patients classified as hypertensive in pre-test (13 [31.0%]) compared with post-test (3 [7.1%]) measurements.

A recent study by Soltani et al. (2019), conducted in hypertensive patients, showed that a regimen of only 8-weeks of two HIIT protocols, including a very short HIIT (30s/30s at 80% of $\dot{V}$O_2__*peak*_, 30 s recovery, ∼40 min total sessions) and a long-duration HIIT (4 min/4 min at 75% of $\dot{V}$O_2__*peak*_, 4 min recovery, ∼42 min total sessions) protocol reduced SBP by −8.1 and −7.6 mmHg, respectively. Although the authors also reported a reduction in blood and plasma viscosity, and in fibrinogen concentration, no exercise substrates or metabolism outcomes were evaluated (Soltani et al., 2019). The present study, albeit with a longer exercise program (16-weeks), but with ≤ 30 min duration of total exercise per session, showed a superior reduction in SBP of Δ−8.70 mmHg in the H-HTN group and Δ−5.11 mmHg in the H-PreHTN group, but also led to relevant clinical changes in the proportion of patients classified as hypertensive in the pre-test (13 [100%]) as compared with post-test (3 [23.0%]) measurements. This suggests that HIIT is a powerful, non-pharmacological therapy against hypertension progression. Along these lines, an interesting parallel effect has been reported by traditional MICT (∼3 days, ∼40 min session, ∼65% heart rate), where a meta-analysis including 30 studies in HTN patients reported SBP/DBP decreases of −6.9 and −4.9, respectively (Fagard and Cornelissen, 2007). However, in comparison to the low-volume implicated by our HIIT protocol (10 min for training, 20 min rest periods), our exercise modality appears more time-efficient as compared to MICT.

More recently, (Olea et al., 2017) reported that after 24 cycling sessions (8 weeks), using a similar HIIT protocol to ours, all 22 hypertensive patients showed a reduction in systolic/diastolic BP (SBP Δ−27 mmHg and DBP Δ−2 mmHg), which changed their diagnosed clinical baseline from hypertensive to normotensive. The authors similarly reported an increase in $\dot{V}$O_2__max_ of Δ + 3.5 mL⋅kg^–1^⋅min^–1^ in hypertensive patients and Δ + 3.4 mL⋅kg^–1^⋅min^–1^ in normotensive subjects at the end of the HIIT program, although no exercise substrate metabolic outcomes were reported. In an classic study (Cade et al., 1984), reported that, in 101/105 hypertensive patients, a 12-week regimen of traditional endurance continuous exercise (walking 2 miles/day with moderate-to-vigorous intensity) led to a significant decrease in SBP which was similar to our research; nevertheless, it is important to mention that in our study more robust effects were observed in SBP and not in DBP, which could be related to improvements in heart physiology rather than a local effect on blood vessels. However, of the 47 patients from the Cade et al. (1984) study who were receiving hypotensive pharmacological therapy during the pre-exercise period, 24 were able to discontinue all medication. Again, no exercise substrate metabolism or cardiorespiratory fitness outcomes were reported by these authors.

In addition to the results presented here, we have previously reported significant decreases in blood pressure and changes in the initial diagnoses of baseline hypertension or prehypertension patients (Cano-Montoya et al., 2016; Álvarez et al., 2018), but our present study is the first to report both blood pressure changes (as a change in clinical diagnosis), exercise substrate metabolism and cardiorespiratory fitness improvements in a hypertensive cohort. These finding demonstrate that HIIT could be considered an effective therapy against hypertension. In this regard, when HTN patients are under hypotensive pharmacotherapy and are also participating of exercise training, there is of great relevance to evaluate regularly the blood pressure in these patients (i.e., the register of before and after the exercise session) and to do the appropriate delivery to the physicians of this inform. This, due to the beneficial of the exercise adaptations can promote fast regulations (i.e., reductions) in their daily hypotensive dose, where physicians adjust these, according with the blood pressure values of each patient. On the other hand, no relationships were observed when testing the association between potential improvements in cardiorespiratory fitness/exercise substrate metabolism with blood pressure improvements (Figure 5). Thus, it is likely that rates of oxidation during exercise conditions or markers of oxidative capacity may not entirely reflect the capacity of obese persons for fatty-acid or CHO metabolism during exercise (Cao et al., 2019). However, there are numerous indications that obesity is associated with a diminished capacity to oxidize fat (van Baak, 1999). In this line, impairments in the ability to mobilize fatty acids from adipose tissue and to oxidize fatty acids in skeletal muscles have been reported in obese subjects during catecholamine stimulation (through β-adrenoceptors; (van Baak, 1999).

In other hand, FATox or CHOox rate in sedentary, obese/hypertensive subjects after chronic exercise training interventions and utilization during exercise is not well documented. Also, in obese individuals the findings are controversial. Kempen et al. (1995) showed changes in body composition, energy expenditure, and substrate utilization in obese women after an 8-week combined diet and exercise training programme compared to diet alone. Furthermore, van Aggel-Leijssen et al. (2001) showed that, in obese men, low-intensity training (40% of $\dot{V}$O_2__max_) resulted in an increased total FATox during MICT, which could be attributed to an increase in non-plasma fatty acid oxidation, whereas HIIT (70% of $\dot{V}$O_2__max_) did not affect total FAT. In contrast, Kanaley et al. (1993) found that a 16-weeks aerobic exercise training programme (45% of $\dot{V}$O_2__max_) did not increase exercise FATox in upper- and lower-body obese women, but rather did increase exercise CHOox. Buemann et al. (1992), in a training study (3 × week, 45 ± 60 min of outdoor running and cycling for 3 ± 4 months) in post-obese women no effect on 24 h RER was shown. In agreement with our findings we speculated that a low-intensity exercise programme may be more effective in increasing FAT/CHOox during peak exercise. This is especially relevant because it has been proposed that low-intensity exercise may be more beneficial in improving FATox during exercise, but that HIIT may be more effective in increasing post-exercise FATox (van Aggel-Leijssen et al., 2001).

Several of the aforementioned studies also reported significant changes in body mass in hypertensive patients after HIIT programs. For example, the study by Olea et al. (2017) reported a change (Δ) of −3.5 kg. However, the other above-mentioned studies did not find changes (Cano-Montoya et al., 2018). In the present study, we found significant body mass changes (Δ−2.79 kg), as well as percentage body fat decreases (Δ−1.38%) in the H-HTN group, together with the aforementioned SBP decreases (Δ−8.70 mmHg) in this group. At 16-week, changes in SBP were negatively correlated to changes in $\dot{V}$O_2__max_ (*R*^2^ = −0.444; *p* = 0.045) only in the H-HTN group. There were no significant changes in by Δ$\dot{V}$O_2__max_ and the ΔDBP levels (Supplementary Figure S1). It is worth noting that a minimum of ∼2 mmHg systolic BP reduction in hypertensive patients is related to a 10% decrease in brain vascular accidents and a 7% decrease in cardiovascular disease (Lewington et al., 2002), suggesting that these results have clinical implications. These changes observed in our study is a significant and clinically relevant finding. In this line, that Kodama et al. (2009) reported that a 1-unit of metabolic equivalents higher level of $\dot{V}$O_2__max_ was associated with a decrement of 13 and 15% in risk of all-cause mortality and cardiovascular disease events, respectively, in healthy men and women.

### Limitations and Strengths

This study has six limitations: (i) this was not a randomized control study, but rather an interventional study with pragmatic applications to evaluate the effectiveness of HIIT in real-life routine practice conditions on blood pressure and cardiorespiratory fitness/exercise substrate metabolism in hypertensive patients; (ii) there was a lack of a true control group, but this was not an aim of the study; (iii) the nutritional habits of participants were not evaluated, but we reminded all participants on a weekly basis to maintain their baseline dietary habits; (iv) the IPAQ score was not incorporated, as this was not an aim of the study, and (v) we did not included vascular function test and (vi) the use of indirect calorimetry method for estimating fat and CHOox, since there are other more accurate methods (i.e., isotope tracers or doubly-labeled water). However, the main strength of our study is that we reported an integral approach of blood pressure changes after a time-efficient exercise training as HIIT, reporting results in mean absolute (mmHg), delta (mmHg), as well as at individual changes. We also included clinical frequently used cut-off points for blood pressure classification to each participant for a better understanding.

## Conclusion

A 16-week HIIT-intervention improved the $\dot{V}$O_2__max_, decreases the body mass and systolic BP especially in the pre-hypertensive and hypertensive patients, independent of changes in exercise substrate metabolism outcomes. Despite slightly greater improvements in some body composition variables or cardiorespiratory fitness, the changes observed in the exercise substrate metabolism were not related to the other BP changes. Because a disturbed muscle FATox or CHOox may be a primary event in the etiology of obesity and/or high blood pressure it is of the utmost importance to know whether, and how, exercise training may compensate for these impairments.

## Data Availability Statement

The raw data supporting the conclusions of this article will be made available by the authors, without undue reservation.

## Ethics Statement

The studies involving human participants were reviewed and approved by The local Ethics Committee reviewed and approved the study protocol (DI18-0043). All participants signed a written informed consent and the study was developed according the tenets of the Declaration of Helsinki. The patients/participants provided their written informed consent to participate in this study.

## Author Contributions

PD-F contributed to the conception, organization and oversight of the study, drafting of the analysis plan, writing of the original manuscript draft, and final approval of the version to be published. FC-N, RM, and DJ-M contributed to measurements in the lab, searching the literature and reviewing the manuscript. CÁ and RR-V contributed to the statistical analyses, writing of the original manuscript draft and final approval of the version to be published. RR-V contributed to critical revision of the manuscript and final approval of the version to be published. RR-V, DA, and MI contributed to data analysis and interpretation, critical manuscript revision and final approval of the version to be published. All authors contributed to the article and approved the submitted version.

## Conflict of Interest

The authors declare that the research was conducted in the absence of any commercial or financial relationships that could be construed as a potential conflict of interest.

## Supplementary Material

FIGURE S1 General correlations (panel **A** and **B**), and by group correlations (panel **C** and **D**) among cardiorespiratory fitness (i.e., by O_2__max_) with blood pressure levels (ΔSBP/ΔDBP), following groups training from baseline to 16-weeks post-exercise. Panel **(A)** denotes the general correlation (i.e., all data) of O_2__max_ with ΔSystolic BP, panel **(B)** denotes general correlation between O_2__max_ with ΔDistolic BP, panel **(C)** denotes by group correlations between O_2__max_ with ΔSystolic BP, and panel **(D)** denotes by group correlations between O_2__max_ with ΔDiastolic BP. Groups are described as (H-CG) HIIT-normotensive control group, (H-PreHTN) HIIT-prehypertensive group, and (H-HTN) HIIT-hypertensive group. β = Standardized coefficients beta and *R*^2^ = R square, coefficient of determination with adjustment for the age, gender and BMI as covariates.
